# Mitochondrial and nuclear gene sequencing confirms the presence of the invasive sea anemone *Diadumene lineata* (Verrill, 1869) (Cnidaria: Actiniaria) in Argentina

**DOI:** 10.7717/peerj.16479

**Published:** 2023-11-27

**Authors:** Ricardo González Muñoz, Daniel Lauretta, María Cielo Bazterrica, Francisco Alejandro Puente Tapia, Agustín Garese, Gregorio Bigatti, Pablo E. Penchaszadeh, Betina Lomovasky, Fabián H. Acuña

**Affiliations:** 1 Laboratorio de Biología de Cnidarios, Facultad de Ciencias Exactas y Naturales, Universidad Nacional de Mar del Plata, Mar del Plata, Buenos Aires, Argentina; 2 Instituto de Investigaciones Marinas y Costeras (IIMyC-CONICET/UNMdP), Mar del Plata, Buenos Aires, Argentina; 3 Laboratorio de Ecosistemas Costeros, Plataforma y Mar Profundo—Malacología, Museo Argentino de Ciencias Naturales “Bernardino Rivadavia”, CONICET, Ciudad Autónoma de Buenos Aires, Argentina; 4 Gabinete de Zooplancton, Instituto Nacional de Investigación y Desarrollo Pesquero-Consejo Nacional de Investigaciones Científicas y Técnicas (INIDEP-CONICET), Mar del Plata, Buenos Aires, Argentina; 5 Laboratorio de Reproducción y Biología Integrativa de Invertebrados Marinos, (LARBIM, IBIOMAR), CONICET, Puerto Madryn, Chubut, Argentina; 6 Universidad Espíritu Santo, Guayaquil, Ecuador; 7 Estación Científica Coiba (Coiba-AIP), Panamá, Panamá

**Keywords:** Argentinian coast, Asexual reproduction, Benthic intertidal fauna, Marine invasive invertebrates, Mitochondrial markers, Nuclear markers, Southwestern Atlantic

## Abstract

**Background:**

*Diadumene lineata* is one of the most widespread sea anemone species worldwide. Although this species has been reported a few times on the Argentine coast since 2004, its identification has traditionally been based on external morphological characteristics, and in most cases no voucher specimens are available to support previous records.

**Methods:**

In this study, we obtained DNA sequences of two mitochondrial markers (12S and 16S) and two nuclear markers (18S and 28S) from specimens of *D. lineata* collected in two locations on the Argentine coast separated by almost 800 km. Additionally, we conducted an analysis of the morphology, as well as the types and size ranges of cnidae, using specimens collected at three different locations along the Argentine coast. Furthermore, since introduced populations of *D. lineata* are presumably ephemeral and only reproduce asexually outside their native range, we examined the internal anatomy of representatives from the Argentine coast for gametogenic tissue as an indication of whether they might be capable of sexual reproduction.

**Results:**

DNA data support our morphological identification, including cnidae analyses, of the specimens as *D. lineata*. Furthermore, all specimens examined were determined to be sterile.

**Discussion:**

Genetic sequence comparisons, phylogenetic reconstruction, and cnidae data support the identification of individuals of *D. lineata* from Mar Chiquita and Garipe Beach, confirming the presence of the species on the Argentine coast using both morphological and molecular tools. The absence of fertile specimens suggests that each sampled population is likely reproducing only by asexual reproduction and possibly composed of clones. The presence of an additional category of longer *p*-mastigophores B2a in the actinopharynx and filaments, as well as holotrichs in the column, is also reported.

**Conclusions:**

For the first time, we have confirmed the presence of *D. lineata* in Argentina through molecular data. Additionally, our findings indicate that the analyzed specimens are sterile, suggesting that this species is not engaging in sexual reproduction in the studied localities. It is crucial to continue monitoring the populations of *D. lineata* along the Argentine coast to assess whether they establish sexual reproduction, expand their distribution range or disappear, or potentially cause any harm to local species or alterations in benthic communities.

## Introduction

Presumably native to Japan, *Diadumene lineata* (Verrill, 1869) is among the sea anemones recognized as a potentially invasive species that has been widely reported in coastal areas of most continents (*e.g.,* Shick & Lamb, 1977; Ting & Geller, 2000; Hancock, Goeke & Wicksten, 2017; Glon et al., 2020; Gimenez, Rivera & Brante, 2022). Its success as an alien species has been facilitated by its ability to withstand wide ranges of temperature and salinity (Shick, 1976; Podbielski et al., 2016; Ryan & Miller, 2019), as well as its ability to reproduce asexually *via* longitudinal fission or pedal laceration (Gollasch & Riemann-Zürneck, 1996; Ting & Geller, 2000; Ryan & Kubota, 2016).

Introduced populations of *D. lineata* are often ephemeral (Stephenson, 1935; Shick, 1976; Dunn, 1982) and mainly consist of clones (Glon et al., 2020). It has been assumed that this species only reproduces asexually outside its native range because the populations studied in the invaded areas were composed of either sterile individuals or only males or females, not both sexes simultaneously (*e.g*., Shick & Lamb, 1977; Minasian, 1982; Ting & Geller, 2000; Glon et al., 2020). However, recent reports have documented the presence of fertile males and females on both the Pacific and Atlantic coasts of the USA (Newcomer, Flenniken & Carlton, 2019; Ryan & Miller, 2019). The occurrence of simultaneously fertile male and female individuals in introduced populations indirectly suggests the possibility of sexual reproduction events taking place in colonized areas, increasing the likelihood of permanent establishment and dispersion of the species in the new area.

**Figure 1:**
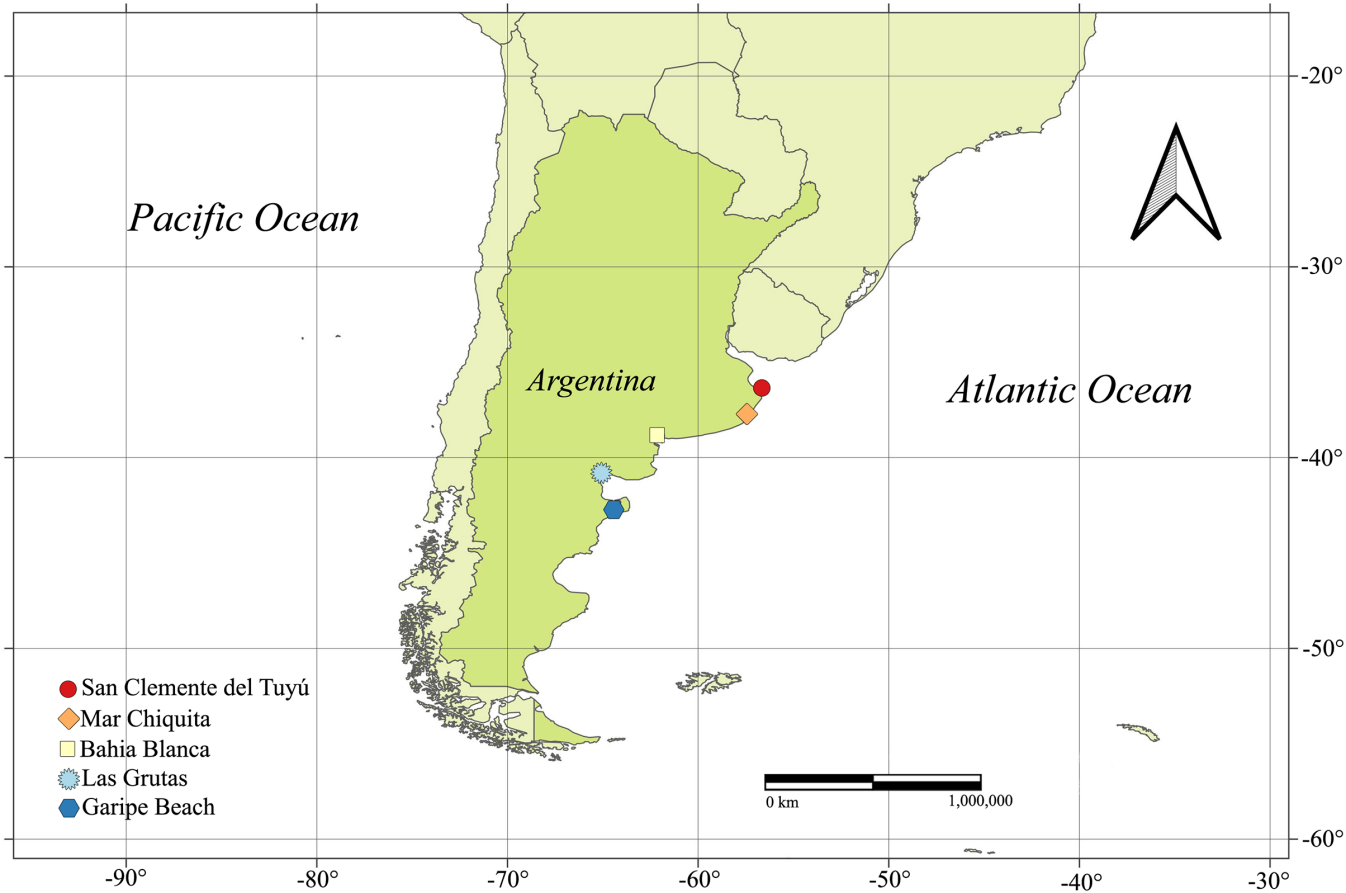
Distribution of *Diadumene lineata* documented along the coast of Argentina.

The first record of *D. lineata* (= *Haliplanella lineata*) in Argentina (Southwestern Atlantic Ocean) dates back to 2004 in the intertidal zone of Las Grutas, Province of Río Negro (Excoffon, Acuña & Zamponi, 2004). The reported specimens did not exhibit the typical color pattern or smooth column (they had a black and vesiculated-like scapus), but this was considered as intraspecific variation (Excoffon, Acuña & Zamponi, 2004). It was later reported in salt marshes in the Bahía Blanca estuary (Buenos Aires Province), where it was found in sediments associated with the roots and stems of *Spartina alterniflora* Loisel, 1807 (Molina et al., 2009). In 2013, it was reported in rocky Patagonian shores by the citizen science program ProyectoSub (https://www.proyectosub.org.ar/, identification made by DL). Furthermore, it was mentioned in rocky Patagonian marshes (Battini & Bortolus, 2020) and in Puerto Pirámides, on the coast of Golfo Nuevo, Province of Chubut, based solely on photographs (iNaturalist, 2022) (Fig. 1). Additionally, its worldwide distribution has recently been modeled, reporting the highest probabilities of occurrence in the northern coastal region of Buenos Aires Province (Gimenez, Rivera & Brante, 2022). No voucher specimens were deposited in any cases, except for the specimens from Bahía Blanca. Unfortunately, probably by logistical constraints, none of these reports included molecular data to support species identification.

*Diadumene lineata* is traditionally recognized by its column color pattern, which is dark green or brown, with longitudinal stripes of orange, yellow, white, or green (Hancock, Goeke & Wicksten, 2017). However, taxonomic identifications based just on external features (*e.g*., color pattern and tentacle number) or internal morphological characteristics (*e.g*., the number of mesenteries, and shape of muscles) can be challenging, especially since specimens derived from asexual reproduction often exhibit irregular arrangements of structures (Atoda, 1973). Therefore, identifications using molecular information would ensure accurate species identification (Beneti et al., 2015).

Correct species identification is crucial when studying invasive species. It represents the minimum requirement for such studies, and species identification based solely on morphological characteristics often requires the expertise of a taxonomist, which may be limited or even unavailable depending on the taxa (Darling & Blum, 2007; Schwindt et al., 2020).

In this study, we obtained DNA sequences of two mitochondrial and two nuclear markers from four specimens of *D. lineata* collected in Argentina. One specimen was collected in Mar Chiquita (Buenos Aires Province) while three specimens were collected in Garipe beach (Chubut Province), separated by 800 km. Additionally, we conducted an analysis of the internal morphology, and the types and size range of cnidae using specimens collected from three coastal locations along the Argentine coast. These analyses aimed to support taxonomic identification by integrating morphological and molecular data to confirm the presence of the invasive sea anemone *D. lineata* in the country. Furthermore, given the presumed ephemeral nature of introduced *D. lineata* populations and their exclusive asexual reproduction outside their native range, we examined the internal anatomy of representatives from the Argentine coast to assess the presence of gametogenic tissue, providing insight into their potential for sexual reproduction.

## Materials and Methods

### Sampling

Eight specimens of putative *D. lineata* were collected in the intertidal zone of Garipe beach (42°35′55.51″S, 64°49′25.94″W), Chubut Province, in November 2013 (austral spring), fifty specimens were collected in the intertidal zone of Mar Chiquita (37°44′30.45″S, 57°24′54.98″W) in April 2021 (austral autumn), and twenty specimens in the coast of San Clemente del Tuyú (36°21′42.10″S, 56°42′45.64″W) in June 2017 (austral winter), both localities in Buenos Aires Province (Fig. 1). Specimens were collected by hand, using a small blunt knife. Individuals from Buenos Aires Province were transferred to the laboratory and kept in an aquarium for photographing their living coloration while those from Chubut Province were photographed both *in situ* and in the laboratory. Some specimens were fixed and preserved in 96% ethanol for molecular studies, but most were anesthetized in 5% MgCl_2_ solution or menthol crystals, then fixed in 7–10% formalin in seawater for several months before being transferred to 70% ethanol for long-term storage. Voucher specimens were deposited in the invertebrate collection of the Museo Argentino de Ciencias Naturales “Bernardino Rivadavia” (MACN) and assigned the following numbers: MACN-In 44051 (Garipe beach, Chubut), MACN-In 44052 (San Clemente del Tuyú, Buenos Aires) and MACN-In 44053 (Mar Chiquita, Buenos Aires).

### Molecular data collection and phylogenetic analysis

Total genomic DNA was extracted from tissue from four specimens (three from Garipe beach and one from Mar Chiquita) following the procedures implemented by Lauretta et al. (2014). We targeted two mitochondrial (12S and 16S rDNA) and two nuclear (18S and 28S rDNA) gene regions. PCR profiles followed those described in Brugler et al. (2018). PCR products were purified and sequenced by Macrogen, Korea. The new sequences from 12S, 16S, 18S and 28S have been deposited in GenBank (Tables 1, S1) and were compared with those from *D. lineata* and other congeners obtained from Genbank (S1). We performed two different analyses of the genetic data. First the sequences were aligned using ClustalW in BioEdit (Hall, 1999) with default parameters, and the Kimura’s two-parameter (K2P) model (Kimura, 1980) of base substitution was used to calculate genetic distances using MEGA11 (Tamura, Stecher & Kumar, 2021). This metric is suitable for sequences with low divergence rates (Nei & Kumar, 2000) and has been employed in calculating distances for other actiniarians (*e.g*., Beneti et al., 2015; González-Muñoz et al., 2015; Grajales & Rodríguez, 2016; Yoshikawa et al., 2022), facilitating comparisons across different groups (Daly et al., 2010). Then, a phylogenetic reconstruction was carried out following the approach implemented by Gusmão, Grajales & Rodríguez (2018) for a similar analysis of *Diadumene* spp. We incorporated our 12S, 16S, and 18S sequences into the matrix from Gusmão, Grajales & Rodríguez (2018), which consists of three mitochondrial gene regions (12S, 16S and COX3) and one nuclear (18S) (S1). The matrix included representatives of the superfamily Metridioidea and used four species of Actinioidea Rafinesque, 1815 as outgroups. The other sequences were obtained from GenBank (see S1 for details). The gene regions were aligned separately using MAFFT (Multiple Alignment using Fast Fourier Transform) ver. 7 (https://mafft.cbrc.jp/alignment/server/) with the E-INS-I strategy, Gap open penalty of 1.53, and default parameters. For the 12S, 16S and 18S sequences, hypervariable regions were removed using Gblocks (Castresana, 2000) with relaxed parameters. The final matrix consisted of 86 taxa and 3,757 total sites. We use a maximum likelihood approach with the IQ-TREE webserver (Trifinopoulos et al., 2016) to infer the phylogenetic relationships. The best substitution model for our data was selected using a different partition per gene (best-fit model according to BIC (Bayesian Information Criterion): TIM2+F+I+G4: 12s, HKY+F+I+G4: 16s, TIM2e+I+G4: 18s, TIM3+F+G4: COX3_1, TIM2e+G4: COX3_2, TPM2u+F+I+G4: COX3_3) (Kalyaanamoorthy et al., 2017). Gaps were treated as missing data in the analyses. Support for each node was calculated using ultrafast bootstrap with 1,000 replicates (Hoang et al., 2018).

**Table 1:** GenBank accession numbers of the taxa included in the genetic distance estimates using the K2P model; new sequences in bold.

Species	12S	16S	18S	28S
*D. lineata* Mar Chiquita	**OP683484**	**OP687084**	**OP688013**	**OP683573**
*D. lineata* 308 Garipe beach	**OP683485**	**OP687085**	**OR395177**	**-**
*D. lineata* 309 Garipe beach	**OP683486**	**OP687086**	**OR395178**	**-**
*D. lineata* 310 Garipe beach	**OP683487**	**OP687087**	**OP688014**	**-**
*Diadumene lineata* (Japan)	JF832965.1	JF832973.1	JF832987.1	JF832998.1
*Diadumene lineata* (USA)	EU190730.1	EU190774.1	EU190860.1	EU190819.1, KJ483107.1, KJ483108.1
*Diadumene lineata* (USA–California)	MT893227.1	–	MT895444.1	MT893020.1
*Diadumene sp*.	JF832960.1	JF832976.1	JF832980.1	JF832990.1
*Diadumene cincta*	EU190725.1	EU190769.1	EU190856.1	EU190814.1, KJ483106.1
*Diadumene leucolena* (Brazil)	KY815042.1	KY815043.1	KY815044.1	KJ483123.1
*Diadumene leucolena*	JF832957.1	JF832977.1	JF832986.1	JF832995.1
*Diadumene manezinha* (Brazil)	KY815045.1	KY815046.1	KY815047.1	–
*Diadumene paranaensis*	–	KT353112.1	–	–

### Morphological analysis

Forty specimens from Mar Chiquita and the 20 specimens from San Clemente del Tuyú were dissected and examined under a Leica stereomicroscope (mod. EZ4-W) to assess the mesenterial arrangement and development of gametogenic tissue. Additionally, serial histological sections of five specimens from Mar Chiquita and two from Garipe beach were made for a more thorough search of gametogenic tissue; paraffin sections 5–7 µm thick were stained with hematoxylin-eosin (Estrada-Flores, Peralta & Rivas, 1982). Measurements of cnidae size ranges were obtained from two specimens from each locality (N = 6). Squash preparations were obtained from small tissue portions (1 mm^3^) of acontia, actinopharynx, mesenterial filaments, column, and tentacles of each individual. For each squash preparation, the length and width of up to 20 undischarged capsules (when possible) of each cnidae type present were randomly measured using microscopy at 1,000× oil immersion. The terminology for cnidae types follows the classification implemented by Gusmão, Grajales & Rodríguez (2018).

## Results

### Comparison of genetic markers

Our analysis of molecular data revealed a high resemblance between our samples and specimens previously identified as *D. lineata* in the existing literature. The comparison of aligned sequences of the 12S and 16S mitochondrial markers showed no variation among individuals from Mar Chiquita and Garipe beach, and *D. lineata* from Japan and the USA (retrieved from GenBank). However, differences were observed when compared with all other congeneric species, such as *Diadumene cincta* Stephenson, 1925, *Diadumene leucolena* (Verrill, 1866), *Diadumene manezinha* Gusmão, Grajales & Rodríguez, 2018, or *Diadumene paranaensis* Beneti et al., 2015. The divergence estimates between *D. lineata* and other species of *Diadumene* varied across these markers, ranging from 0.57‒0.96% for 12S and 1.49‒2.10% for 16S (Tables 2, S2). The 18S nuclear marker sequence from the Garipe beach specimen showed variations compared to those from Mar Chiquita, with 11 substitutions and three ambiguous sites. The positions of these substitutions were based on the sequence of the *Diadumene lineata* voucher SaCr1A small subunit ribosomal RNA gene (GenBank accession number: MT895444.1, of 1,785 base pairs in length). The substitutions consisted of three transitions: (T to C, at positions 912 and 1176; C to T, at position 1710) and eight transversions (G to C, at positions 767 and 1056; T to A, at positions 882, 1061, 1123; G to C, at position 993; G to T, at position 1049; C to G, at position 1164). The three sites with ambiguities were T to Y, at positions 1277 and 1306; G to R, at position 1296. For the 18S marker, the intraspecific divergences among the sequences of *D. lineata* varied from 0.00 to 0.74%, whereas between *D. lineata* and the other species of *Diadumene*, they ranged from 4.25 to 5.11% (Tables 2, S2). The sequences of the 28S nuclear marker could only be obtained from specimens from Mar Chiquita, but no variation was found between these sequences compared to others of *D. lineata* from GenBank. However, similarly to the 18S marker, there was an intraspecific divergence of 0.45% in a sequence from California, USA (Table 2). The genetic distance calculated for the 28S nuclear marker between *D. lineata* and other species of *Diadumene* ranges from 9.28‒13.74% (Tables 2, S2).

**Table 2:** Estimated divergence (K2P, expressed as a percentage) of *Diadumene lineata* compared to other *Diadumene* species, based on the comparison of sequences from the four molecular markers used.

	12S	16S	18S	28S
*D. lineata* Mar Chiquita	0	0	0	0
*D. lineata* 308 Garipe beach	0	0	0	–
*D. lineata* 309 Garipe beach	0	0	0	–
*D. lineata* 310 Garipe beach	0	0	0.74	–
*D. lineata* (USA)	0	0	–	0
*D. lineata* (Japan)	0	0	–	0
*D. lineata* MT893227.1/MT893020.1	0	–	–	0.45
*Diadumene* sp.	0.96	1.79	4.25–5.03	11.37
*Diadumene cinta*	0.96	1.79	4.25–5.03	12.94–13.74
*Diadumene manezinha*	0.57	1.79	4.32–5.11	–
*Diadumene leucolena*	0.57	1.49	4.25–5.04	9.28–9.80
*Diadumene paranaensis*	–	2.10	–	–

### Molecular phylogenetic analysis

**Figure 2:**
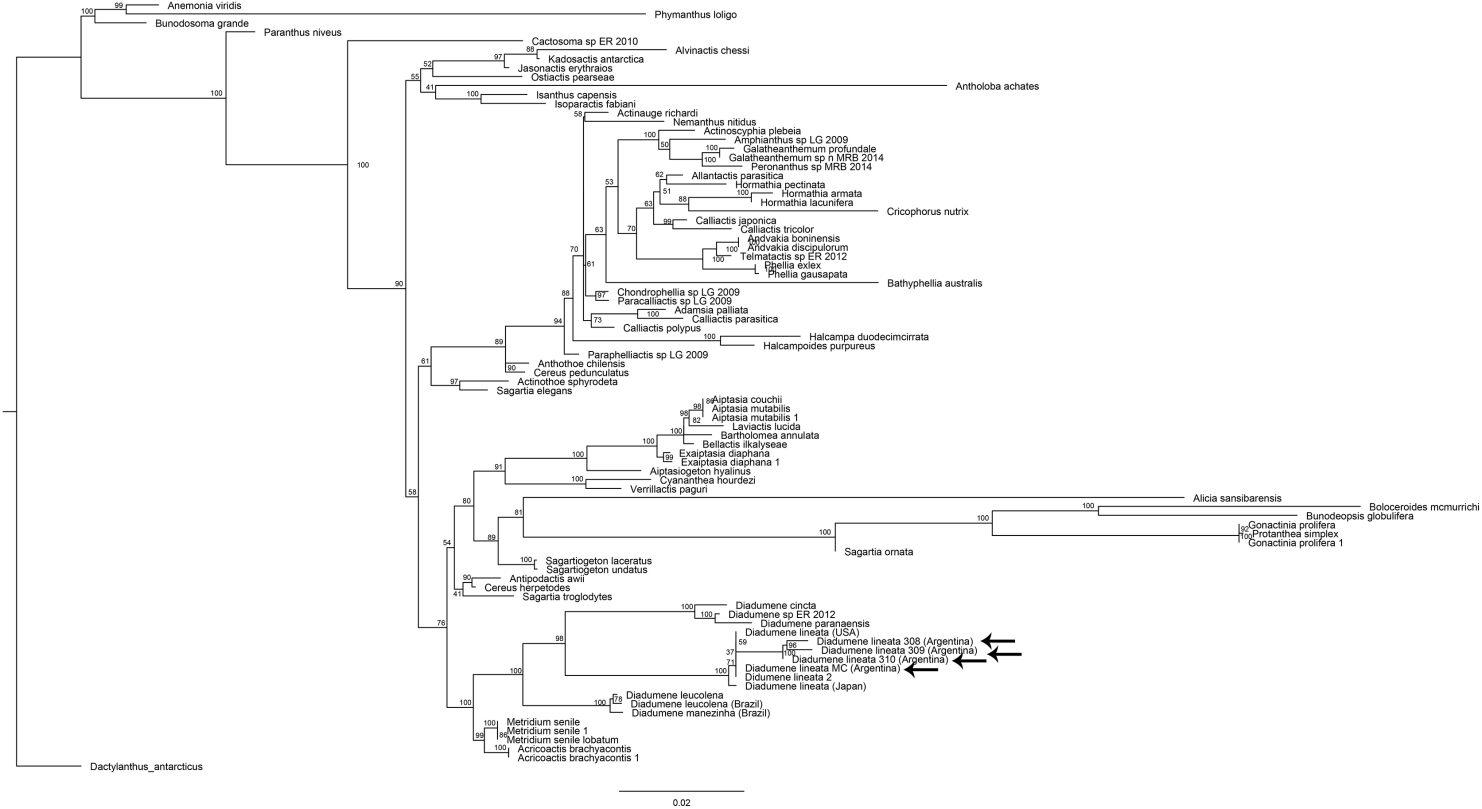
Maximum-likelihood tree from combined analysis of 12S, 16S, COX3, and 18S sequences. Branch numbers indicate Ultrafast Bootstrap values. Arrows point to *D. lineata* specimens collected in Argentina.

The phylogenetic reconstruction recovered the genus *Diadumene* as monophyletic with high support (100%) and composed of three major clades (Fig. 2): one clade formed by *D. cincta*, *Diadumene* sp., and *D. paranaensis* (100%), a second clade by *D. leucolena* and *D. manezinha* (100%), and a third clade by *D. lineata* from the USA, Japan, and the Argentine samples (100%). Then, Argentine samples were grouped within the *D. lineata* clade (Fig. 2). Similarly to Larson (2016) and Gusmão, Grajales & Rodríguez (2018), we recovered the clade Metridina of Rodríguez et al. (2012) with high support (100%), which is composed of Diadumenidae as the sister group to a clade with members of Metridiidae Carlgren, 1893 and Acricoactinidae Larson, 2016.

### Morphological examination

**Figure 3:**
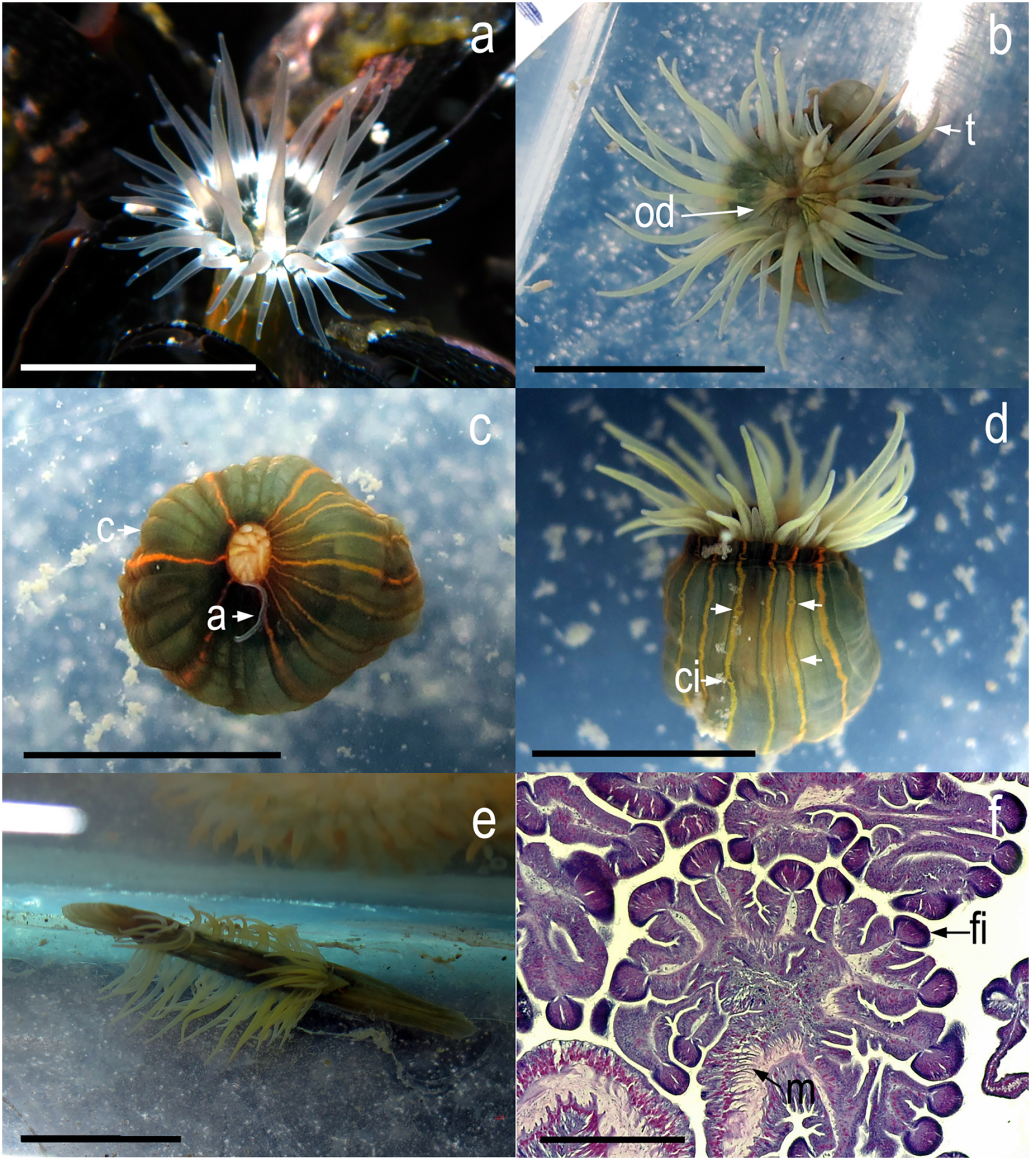
*In vivo* images of *Diadumene lineata* from Argentina. (A) *in situ*, top view, detail of distal part of the column and oral disc (specimen from Garipe beach); (B) top view, detail of oral disc (specimen from Mar Chiquita); (C) top view, oral disc and tentacles contracted; (D) lateral view, detail of column. Arrows point to cinclides; (E) top view, specimen splitting by longitudinal fission in aquarium; (F) histological transversal section, detail of mesenteries with no trace of gametes. Abbreviations.- a, acontia; c, column; ci, cinclides; fi, mesenteric filaments; m, mesenterial retractor muscles; od, oral disc; t, tentacles. Scale bars.-a-e: 5 mm; f: 200 µm.

**Figure 4:**
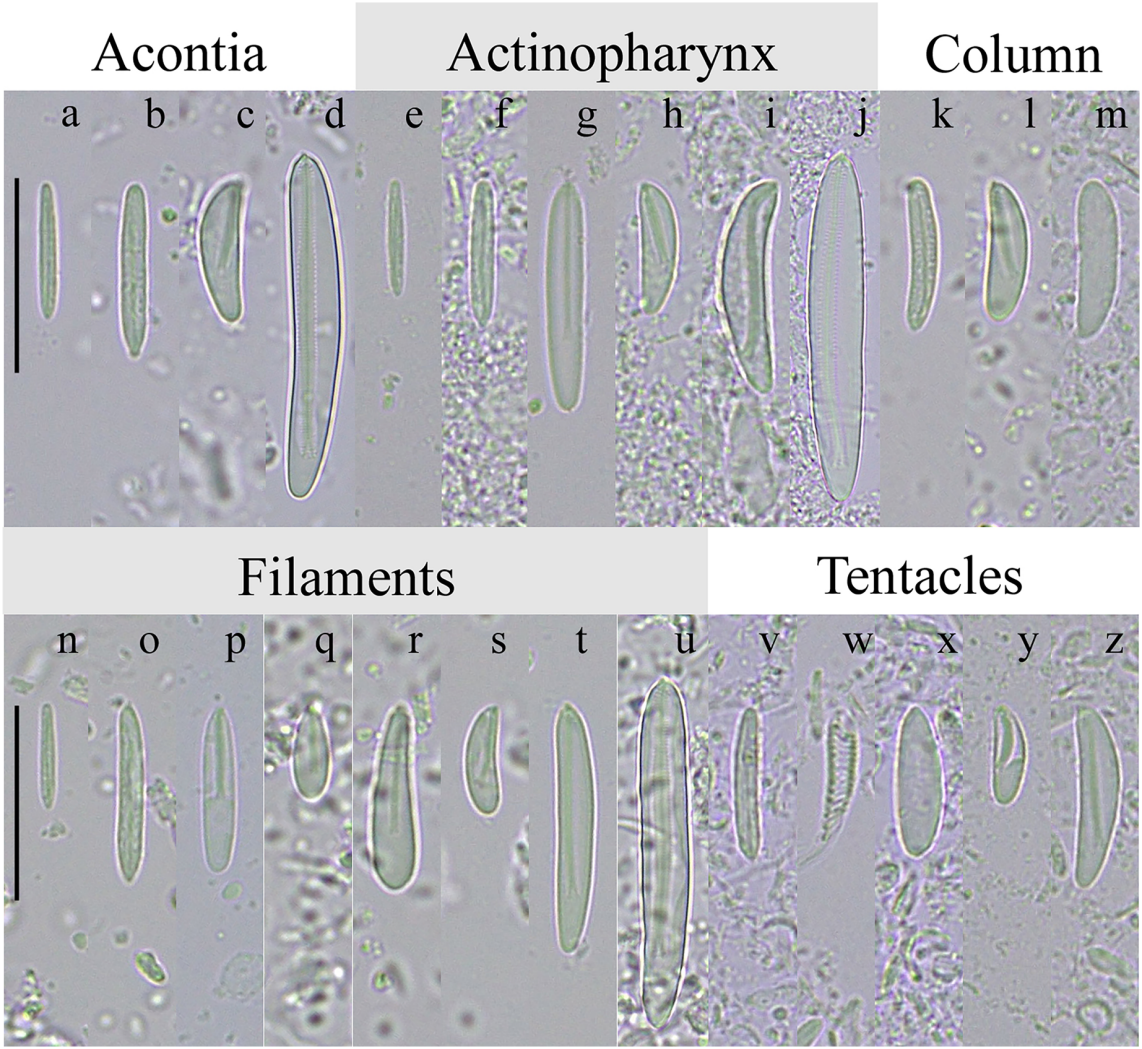
Cnidae of *Diadumene lineata* from Argentina. Notes: (a, e, n) small basitrichs; (b, f, k, o, v) basitrichs; (c, h, l, s, y) small *p*-mastigophores B2a; (d, j, u) large *p*-mastigophores B2a; (i, t, z) medium-size *p*-mastigophores B2a; (g, p) *p*-mastigophores A; (q, r) *p*-mastigophores B1; (m, x) holotrichs; (w) spirocysts. Scale bars: a-c, e-I, k-t, v-z: 20 µm; d, j, y: 14 µm.

Specimens examined showed the external morphology characteristic of *D. lineata*: tentacles relatively large, thin, smooth without any structures, tapered to a point, grayish or grayish-green and translucent (Figs. 3A, 3B, 3D and 3E); oral disc light-brown or light green, translucent (Fig. 3A); column dark-green with orange stripes on scapus from margin to the limbus (Figs. 3A–3D); cinclids clearly visible along the orange stripes (Fig. 3D); white acontia emerging from the mouth or the cinclids when contracted (Fig. 3C). No gametogenic tissue was observed in any of the 60 dissected specimens, nor in those individuals studied with histological sections (Fig. 3F). In addition, the mesenteries presented an irregular arrangement in every specimen examined, with weak and diffuse retractor muscles (Fig. 3F). The cnidom of our specimens of *D. lineata* was composed of spirocysts, basitrichs, holotrichs, and microbasic *p*-mastigophores A, B1, and B2a (Fig. 4). However, not all types of cnidocysts were found in every analyzed specimen, and in some cases, their abundance was also dissimilar (Table 3).

**Table 3:** Comparison of the types and size ranges of cnidae among specimens from the three locations on the Argentine coast.

Categories	San Clemente	X ± SD	S	N	Mar Chiquita	X ± SD	S	N	Garipe beach	X ± SD	S	N
**ACONTIA**												
Small basitrichs	7.9–13.2 × 1.4–2.2	11.2 ± 1.4 × 1.9 ± 0.2	17	2/2	10.3–14.2 × 1.8–2.2	12.0 ± 1.0 × 1.9 ± 0.1	25	2/2	–	–	–	–
Basitrichs	14.7–19.1 × 2.1–3.0	16.7 ± 1.0 × 2.6 ± 0.2	40	2/2	14.8–19.2 × 2.4–3.0	16.8 ± 1.1 × 2.6 ± 0.2	40	2/2	13.6–17.4 × 1.7–2.2	16.1 ± 1.3 × 1.8 ± 0.2	6	1/2
Small p-mastigophores B2a	12.0–18.9 × 2.7–3.7	16.3 ± 1.98 × 3.2 ± 0.3	18	2/2	14.8–19.7 × 3.0–4.1	17.2 ± 1.3 × 3.5 ± 0.3	20	2/2	11.9 × 3.6	–	1	1/2
Large p-mastigophores B2a	38.1–52.8 × 4.9–7.6	47.3 ± 3.4 × 5.8 ± 0.7	21	2/2	44.6–55.9 × 6.0–9.4	50.6 ± 3.1 × 7.4 ± 0.6	21	2/2	36.7–48.6 × 4.8–6.7	44.5 ± 3.1 × 5.8 ± 0.5	20	1/2
**ACTINOPHARYNX**												
Small basitrichs	9.7–14.6 × 1.6–2.6	13.1 ± 1.5 × 2.1 ± 0.3	11	2/2	9.9–14.0 × 1.6–2.5	11.7 ± 1.0 × 2.0 ± 0.3	23	2/2	–	–	–	–
Basitrichs	15.4–18.7 × 2.1–2.9	16.8 ± 1.0 × 2.6 ± 0.2	20	1/2	14.8–21.9 × 2.1–3.3	17.3 ± 1.6 × 2.7 ± 0.3	26	2/2	15.5–24.0 × 1.5–2.6	18.1 ± 3.3 × 2.0 ± 0.4	8	2/2
p-mastigophores A	–	–	–	–	21.2–21.7 × 3.5–3.6	21.4 ± 0.4 × 3.6 ± 0.1	2	1/2	21.8–24.9 × 2.9–4.4	23.3 ± 1.1 × 3.6 ± 0.5	9	2/2
p-mastigophores B2a I	10.4–13.9 × 3.0–3.8	11.9 ± 1.2 × 3.4 ± 0.3	7	1/2	10.5–13.8 × 3.0–4.0	11.6 ± 1.3 × 3.6 ± 0.4	5	1/2	10.8–12.3 × 2.7–3.0	11.6 ± 1.1 × 2.9 ± 0.2	2	1/2
p-mastigophores B2a II	17.2–35.0 × 2.5–4.7	23.5 ± 4.1 × 3.7 ± 0.4	40	2/2	16.0–29.2 × 3.1–4.8	22.5 ± 2.7 × 3.8 ± 0.4	40	2/2	21.8–26.2 × 3.0–4.0	24.2 ± 1.5 × 3.4 ± 0.3	15	2/2
p-mastigophores B2a III	41.5–53.4 × 5.0–7.5	47.6 ± 3.0 × 6.2 ± 0.5	25	2/2	42.6–47.4 × 8.3–8.4	47.4 ± 6.8 × 8.4 ± 0.2	2	1/2	28.2–50.6 × 3.6–6.4	39.4 ± 9.3 × 5.1 ± 1.0	10	2/2
**COLUMN**												
Basitrichs I	15.3–18.9 × 2.3–3.5	17.0 ± 0.7 × 2.9 ± 0.3	22	2/2	12.3–15.9 × 2.5–3.5	14.3 ± 0.9 × 3.0 ± 0.2	40	2/2	12.0–18.1 × 1.9–2.9	16.1 ± 1.5 × 2.4 ± 0.3	29	2/2
p-mastigophores B2a I	9.5–18.9 × 3.3–5.0	15.6 ± 2.1 × 4.1 ± 0.3	40	2/2	11.2–16.1 × 3.3–4.7	13.5 ± 1.0 × 3.9 ± 0.3	40	2/2	11.7–15.4 × 2.7–3.9	13.4 ± 1.2 × 3.3 ± 0.4	8	2/2
Holotrichs	13.4–20.0 × 3.7–6.2	17.3 ± 1.7 × 5.3 ± 0.7	15	2/2	12.3–18.7 × 4.1–6.1	16.4 ± 1.5 × 5.1 ± 0.5	23	2/2	14.7–18.9 × 2.8–5.7	16.5 ± 1.2 × 4.2 ± 0.8	16	2/2
**FILAMENTS**												
Small basitrichs	10.5–12.7 × 1.5–2.2	11.4 ± 0.6 × 1.8 ± 0.2	15	1/2	9.2–11.8 × 1.5–2.6	10.4 ± 0.7 × 1.8 ± 0.2	40	2/2	–	–	–	–
Basitrichs	17.0–19.0 × 2.1–2.3	18.0 ± 1.4 × 2.2 ± 0.2	2	1/2	16.0–20.6 × 2.2–2.9	17.6 ± 1.1 × 2.6 ± 0.2	20	1/2	19.4–19.6 × 2.1–3.0	19.5 ± 0.1 × 2.4 ± 0.5	3	1/2
p-mastigophores A	16.7 × 3.4	–	1	1/2	–	–	–	–	–	–	–	–
p-mastigophores B1 I	9.6–11.1 × 3.7–5.7	10.6 ± 0.4 × 4.6 ± 0.6	20	1/2	8.6–11.2 × 3.1–4.0	10.1 ± 0.6 × 3.6 ± 0.3	17	2/2	11.9 × 3.6	–	1	1/2
p-mastigophores B1 II	15.6–19.6 × 4.5–6.90	17.7 ± 1.1 × 5.4 ± 0.7	20	1/2	16.0–19.2 × 4.3–5.4	17.4 ± 1.2 × 4.6 ± 0.3	11	2/2	–	–	–	–
p-mastigophores B2a I	9.9–17.6 × 2.7–4.4	12.6 ± 2.2 × 3.4 ± 0.5	20	1/2	9.3–15.8 × 2.6–4.0	12.4 ± 1.4 × 3.2 ± 0.3	31	2/2	11.1–15.0 × 2.7–3.0	13.1 ± 2.8 × 2.9 ± 0.2	2	2/2
p-mastigophores B2a II	22.7–27.2 × 3.0–4.0	25.0 ± 1.4 × 3.6 ± 0.2	20	1/2	19.7–29.1 × 3.1–5.1	26.1 ± 2.0 × 3.8 ± 0.4	40	2/2	21.9–32.4 × 2.9–5.2	24.6 ± 2.8 × 3.5 ± 0.5	17	2/2
												
p-mastigophores B2a III	48.5–60.3 × 6.1–8.2	54.2 ± 2.8 × 7.0 ± 0.7	16	1/2	46.6–55.6 × 6.4–10.4	51.3 ± 2.0 × 7.5 ± 0.8	22	2/2	41.9–48.9 × 4.0–5.9	45.8 ± 2.0 × 5.3 ± 0.4	20	1/2
**TENTACLES**												
Basitrichs I	14.3–19.6 × 2.–3.1	17.4 ± 1.1 × 2.4 ± 0.2	39	2/2	12.6–16.6 × 2.0–2.9	14.8 ± 0.9 × 2.4 ± 0.2	40	2/2	13.0–23.9 × 1.9–2.5	19.1 ± 4.6 × 2.3 ± 0.2	5	2/2
Spirocysts	13.6–21.4 × 3.0–5.0	16.8 ± 2.0 × 3.8 ± 0.5	20	2/2	11.8–21.2 × 2.7–5.8	16.5 ± 2.8 × 3.7–0.8	40	2/2	13.3–23.5 × 2.1–3.8	18.8 ± 2.3 × 3.0 ± 0.4	40	2/2
Holotrichs	–	–	–	–	14.7–18.3 × 3.7–5.1	16.6 ± 1.1 × 4.4 ± 0.4	20	1/2	11.2–18.5 × 2.9–3.9	15.5 ± 2.9 × 3.4 ± 0.4	5	1/2
p-mastigophores B2a I	9.5–17.6 × 2.9–4.0	15.0 ± 2.0 × 3.4 ± 0.3	24	2/2	7.7–11.3 × 2.4–3.4	10.0 ± 0.7 × 3.0 ± 0.2	40	2/2	11.9–14.8 × 2.2–3.5	13.1 ± 1.0 × 2.8 ± 0.4	13	2/2
p-mastigophores B2a II	18.2–24.3 × 2.7–3.9	21.2 ± 1.2 × 3.3 ± 0.3	40	2/2	17.1–25.6 × 3.1–4.5	20.5 ± 1.7 × 3.8 ± 0.3	40	2/2	19.4–24.5 × 2.8–4.0	22.3 ± 1.1 × 3.4 ± 0.4	24	2/2

## Discussion

Genetic sequence comparisons support the identification of individuals from Mar Chiquita and Garipe Beach as *Diadumene lineata*, and thus their presence on the Argentine coast is confirmed by molecular tools. The lack of variation among mitochondrial sequences of *D. lineata* (*i.e*. 12S and 16S rDNA) is consistent with the slow rates of mitochondrial sequence evolution observed in many anthozoan groups (Shearer et al., 2002). However, intra-specific variations among mitochondrial sequences have been reported for some species of actiniarians. For instance, González-Muñoz et al. (2015) found two haplotypes of the 12S marker in *Phymanthus crucifer* (Le Sueur, 1817), which differ by a single substitution within an 824 bp fragment (K2P distance = 0.1215%). Furthermore, Grajales & Rodríguez (2016) reported intra-specific variations in the mitochondrial markers 12S and 16S for several species within Aiptasiidae Carlgren, 1924. In their study, genetic distances calculated by the K2P model ranged between 0.002 and 0.052 for 12S, and between 0.0003 and 0.003 for 16S (Grajales & Rodríguez, 2016). The values of intra-specific divergence for mitochondrial markers described in both previous examples are much lower than the divergence ranges observed in the present study between *D. lineata* and other species of *Diadumene* (*i.e*., 0.57–0.96% for 12S, and 1.49–2.10% for 16S). Therefore, although the absence or low divergence often hinders the recognition of differences among populations, it is useful for distinguishing genetic differences between congeneric species, at least for the studied species of *Diadumene*.

For the nuclear marker 18S, the intra-specific variation (ranging from 0.00 to 0.74%) is much smaller compared to the inter-specific variation (ranging from 4.25 to 5.11%). Therefore, we consider that the variations among the *D. lineata* sequences represent intra-specific variability (Table 2). However, these results also suggest that this marker could exhibit some variability among populations of *D. lineata*. This supports the idea that nuclear markers can also show intra-specific variability in sea anemones, as reported in other studies (*e.g*., Grajales & Rodríguez, 2016; Brugler et al., 2018).

The cnidae observed in the examined specimens of *D. lineata* largely agrees with those described by Hand (1955) and Excoffon, Acuña & Zamponi (2004) for this species, both in its composition and in the size ranges of the cnidae. However, the examined specimens exhibit an additional category of *p*-mastigophores B2a in the actinopharynx and filaments, which are longer (*i.e*., 41.9–60.3 and 28.2–53.4 µm, respectively) compared to those reported by Hand (1955) (*i.e*., 18.5–25.0 and 21.5–27.0 µm, respectively) and Excoffon, Acuña & Zamponi (2004) (20–40 µm in the filaments). Nevertheless, the use of different nomenclatures and the absence of images of cnidae types in these previous studies make a proper comparison difficult. Additionally, the presence of holotrichs in the column is reported. A more detailed study on the variation of types and sizes of cnidae among *D. lineata* specimens from distinct populations is required.

The fact that no trace of gametogenic tissue development was found in the specimens examined suggests that the population was sterile and not capable of sexual reproduction, at least at the time of collection (austral autumn, winter, and spring). Therefore, it is most likely that all the individuals examined are clones due to their high capacity for asexual reproduction. As Stephenson (1935) and Shick & Lamb (1977) reported, we observed a specimen from Mar Chiquita that quickly began to divide longitudinally, which could support this hypothesis (Fig. 3E). If these individuals are indeed clones, then it might be expected that after a period of rapid colonization, the population would eventually decline or individuals would become rare, as has been documented in other locations (*e.g*., Shick, 1976; Shick & Lamb, 1977). Molina et al. (2009) suggested that there is a stable population of *D. lineata* in the Bahía Blanca estuary, based on their estimation of population density detecting higher abundances in the summer months; however, it was unclear whether this population was made up of clones or even if included individuals capable of sexual reproduction.

According to Fukui (1995), the peak of sexual reproduction of *D. lineata* in Japan is during the boreal summer (from July to August in the northern hemisphere) and involves the release of gametes. Spermatogenesis starts in late fall when small cysts occur in the mesoglea and develop from winter to late spring. The growth stage of gametes occurs during and after April, and mature cysts could be observed in mid-summer (Fukui, 1995). Our specimens examined from Mar Chiquita and San Clemente del Tuyú were collected in April and June, respectively (during the austral autumn and winter, respectively), which falls outside the documented period of sexual reproduction for this species. Conversely, the specimens from Garipe Beach were collected in late spring, when gametogenesis should be present. However, the implications that the different seasons of the year may have in the northern and southern hemispheres, such as the change in temperature regime among one of the many environmental factors that may be involved in the sexual reproduction of this species, are unknown.

*Diadumene lineata* is one of the four species of sea anemones currently considered in the official list of exotic species of Argentina (Ministry of Environment and Sustainable Development of Argentina, 2022), along with *Aulactinia reynaudi* (Milne Edwards, 1857), currently *Bunodactis reynaudi*, *Oulactis muscosa* (Drayton in Dana, 1846) and *Boloceroides mcmurrichi* (Kwietniewski, 1898). Additionally, Excoffon & Zamponi (1999) documented the occurrence of *Sagartia troglodytes* (Price in Johnston, 1847), currently *Cylista troglodytes*, on the coast of Mar del Plata, a species native to European waters. However, there have been no reports of its presence (nor of *B. mcmurrichi*) in Argentina since then. It has also been demonstrated through molecular tools that the specimens previously identified as *A. reynaudi* belong to the local species *Aulactinia marplatensis* (Acuña et al., 2007). Furthermore, ongoing research aims to verify the uncertain presence of *O. muscosa* and *B. mcmurrichi* in Argentina. Moreover, the official list of exotic species does not include *Metridium senile* (Linnaeus, 1761), which is native to the northern hemisphere (Glon et al., 2020), has previously been reported in the country (*e.g*., Riemann-Zürneck, 1975; Martin et al., 2015), and is recognized as one of the most widely distributed invasive sea anemone species worldwide. Therefore, we consider only two sea anemones as potentially invasive species in Argentina: *D. lineata*, present since at least 1999 (Excoffon, Acuña & Zamponi, 2004) and *M. senile*, present in Argentina since at least 1966 (Riemann-Zürneck, 1975).

According to Schwindt et al. (2020), the most probable vectors for the introduction of *D. lineata* in Argentina are through ship fouling or ballast water. The arrival of this species may be linked to international shipping traffic, with the port of Mar del Plata being a likely initial point of entry. Mar del Plata has a significant port in Argentina, serving both national fishing activities and housing the country’s most important marina (Castro et al., 2021). In fact, Mar del Plata and its surrounding areas have the highest number of reported introduced species (Schwindt et al., 2020), establishing it as a hotspot for invasive marine species (Castro et al., 2021). It is plausible that *D. lineata* has subsequently spread to other coastal areas, potentially facilitated by recreational vessels, as suggested by Castro et al. (2021) in their detailed study on potential routes of introduction and distribution of invasive ascidian species in the country.

Invasive anemones could harm native benthic communities, as recently observed in Chile with the spread of *M. senile* (Häussermann et al., 2022), but the impact of *D. lineata* populations on colonized environments remains uncertain. However, a report from the United Kingdom indicates that this species primarily preys on small crustaceans and may consume bivalve larvae of commercially important species like oysters and mussels, affecting diversity and local fisheries (Wood et al., 2022). *Diadumene lineata* could compete for space and resources with local species that naturally occupy similar niches along the southern coasts of the Buenos Aires province, such as *Anthothoe chilensis* (Lesson, 1830) or *Tricnidactis errans* De Oliveira Pires, 1987. On the other hand, it has been documented that *D. lineata* is heavily preyed upon by nudibranchs like *Aeolidia papillosa* (Linnaeus, 1761) (Shick & Lamb, 1977), similar to local reports of mollusks from the genera *Spurilla* Bergh, 1864 and *Pleurobranchaea* Leue, 1813 feeding on local species of sea anemones in Argentina (Garese et al., 2012; Bökenhans et al., 2018). Therefore, continuous monitoring of *D. lineata* populations in Argentina is crucial to observe whether they establish and reproduce, expand their distribution, decline, or potentially cause harm to local species or alterations in benthic communities.

## Conclusions

We confirm, for the first time, the presence of *Diadumene lineata* in Argentina using mitochondrial and nuclear genetic markers and expand the northernmost distribution limits to include the localities of Mar Chiquita and San Clemente del Tuyú. Furthermore, because the specimens analyzed are sterile (and possibly clones), we propose that this species is not currently undergoing sexual reproduction in the studied localities, at least within the observed timeframe. Therefore, systematic population monitoring over the years is necessary to asses and track the reproductive cycle of *D. lineata* along the Argentinean coast, its spread and potential effects on native communities.

## Supplemental Information

10.7717/peerj.16479/supp-1 Supplemental Information 1 Taxa included in this study, with GenBank accession numbers. New data in bold.

10.7717/peerj.16479/supp-2 Supplemental Information 2 K2P model for 12S, 16S, 18S and 28S.

## Additional Information and Declarations

### Data Availability

The following information was supplied regarding data availability: The data are available in the article and the new sequences are available at GenBank: OP683484, OP687084, OP688013, OP683485, OP687085, OR395177, OP683486, OP687086, OR395178, OP683487, OP687087, OP688014, OP683573.

## References

[ref-4] Atoda K (1973). Pedal laceration of the sea anemone, *Haliplanella luciae*. Publications of the Seto Marine Biological Laboratory.

[ref-5] Battini N, Bortolus A (2020). A major threat to a unique ecosystem. Frontiers in Ecology and the Environment.

[ref-6] Beneti JS, Stampar SN, Maronna MM, Morandini AC, Da Silveira FL (2015). A new species of *Diadumene* (Actiniaria: Diadumenidae) from the subtropical coast of Brazil. Zootaxa.

[ref-7] Brugler MR, González-Muñoz RE, Tessler M, Rodríguez E (2018). An EPIC journey to locate single-copy nuclear markers in sea anemones. Zoologica Scripta.

[ref-8] Bökenhans V, Fernández Alfaya JE, Bigatti G, Averbuj A (2018). Diet of the invasive sea slug *Pleurobranchaea maculata* in Patagonian coastal waters. New Zealand Journal of Zoology.

[ref-10] Castresana J (2000). Selection of conserved blocks from multiple alignments for their use in phylogenetic analysis. Molecular Biology and Evolution.

[ref-11] Castro KL, Battini N, Giachetti CB, Trovant B, Abelando M, Basso NG, Schwindt E (2021). Early detection of marine invasive species following the deployment of an artificial reef: integrating tools to assist the decision-making process. Journal of Environmental Management.

[ref-13] Daly M, Gusmão L, Reft AJ, Rodríguez E (2010). Phylogenetic signal in mitochondrial and nuclear markers in sea anemones (Cnidaria, Actiniaria). Integrative and Comparative Biology.

[ref-14] Darling J, Blum M (2007). DNA-based methods for monitoring invasive species: a review and prospectus. Biological Invasions.

[ref-15] Dunn DF (1982). Sexual reproduction of two intertidal sea anemones (Coelenterata: Actiniaria) in Malaysia. Biotropica.

[ref-16] Estrada-Flores E, Peralta L, Rivas P (1982). Manual de técnicas histológicas.

[ref-17] Excoffon AC, Acuña FH, Zamponi MO (2004). Presence of *Haliplanella lineata* (Verrill, 1869) (Actiniaria, Haliplanellidae) in the Argentine sea and the finding of anisorhize haploneme cnidocyst. Physis.

[ref-18] Excoffon AC, Zamponi MO (1999). *Sagartia troglodytes* (Price, 1847) (Cnidaria: Sagartiidae) from the south-western Atlantic Ocean and the first evidence of spermatophores in sea anemones. Acta Adriatica.

[ref-19] Fukui Y (1995). Seasonal changes in testicular structure of the sea anemone *Haliplanella lineata* (Coelenterata: Actiniaria). Invertebrate Reproduction & Development.

[ref-20] Garese A, García-Matucheski S, Acuña FH, Muniain C (2012). Feeding behavior of *Spurilla* sp. (Mollusca: Opisthobranchia) with a description of the kleptocnidae sequestered from its sea anemone prey. Zoological Studies.

[ref-22] Gimenez LH, Rivera RJ, Brante A (2022). One step ahead of sea anemone invasions with ecological niche modeling: potential distributions and niche dynamics of three successful invasive species. Marine Ecology Progress Series.

[ref-23] Glon H, Daly M, Carlton JT, Flenniken MM, Currimjee Z (2020). Mediators of invasions in the sea: life history strategies and dispersal vectors facilitating global sea anemone introductions. Biological Invasions.

[ref-24] Gollasch S, Riemann-Zürneck K (1996). Transoceanic dispersal of benthic macrofauna: *Haliplanella luciae* (Verrill, 1898) (Anthozoa, Actiniaria) found on a ship’s hull in a shipyard dock in Hamburg Harbour. Germany Helgolander Meeresuntersuchungen.

[ref-25] González-Muñoz R, Simões N, Mascaró M, Tello-Musi JL, Brugler MR, Rodríguez E (2015). Morphological and molecular variability of the sea anemone *Phymanthus crucifer* (Cnidaria, Anthozoa, Actiniaria, Actinoidea). Journal of the Marine Biological Association of the United Kingdom.

[ref-26] Grajales A, Rodríguez E (2016). Elucidating the evolutionary relationships of the Aiptasiidae, a widespread cnidarian-dinoflagellate model system (Cnidaria: Anthozoa: Actiniaria: Metridioidea). Molecular Phylogenetics and Evolution.

[ref-27] Gusmão LC, Grajales A, Rodríguez E (2018). Sea anemones through X-rays: visualization of two species of *Diadumene* (Cnidaria, Actiniaria) using micro-ct. American Museum Novitates.

[ref-28] Hall TA (1999). BioEdit: a user-friendly biological sequence alignment editor and analysis program for windows 95/98/NT. Nucleic Acids Symposium Series.

[ref-29] Hancock ZB, Goeke JA, Wicksten MK (2017). A sea anemone of many names: a review of the taxonomy and distribution of the invasive actiniarian *Diadumene lineata* (Diadumenidae), with records of its reappearance on the texas coast. ZooKeys.

[ref-30] Hand C (1955). The sea anemones of Central California. Part III. The acontiarian anemones. Wasmann Journal of Biology.

[ref-32] Häussermann V, Molinet C, Díaz Gómez M, Försterra G, Henríquez J, Espinoza Cea K, Matamala Ascencio T, Hüne M, Cárdenas C, Glon H, Barahona Toledo N, Subiabre Mena D (2022). Recent massive invasions of the circumboreal sea anemone *Metridium senile* in North and South Patagonia. Biological Invasions.

[ref-31] Hoang DT, Chernomor O, Haeseler A, Minh BQ, Vinh LS (2018). UFBoot2: improving the ultrafast bootstrap approximation. Molecular Biology and Evolution.

[ref-33] iNaturalist (2022). iNaturalist research-grade observations. iNaturalist.org. Occurrence dataset. https://www.gbif.org/occurrence/3416092598.

[ref-34] Kalyaanamoorthy S, Mihn BQ, Wong TKF, Haeseler A, Jermiin LS (2017). ModelFinder: fast model selection for accurate phylogenetic estimates. Nature Methods.

[ref-35] Kimura M (1980). A simple method for estimating evolutionary rates of base substitutions through comparative studies of nucleotide sequences. Journal of Molecular Evolution.

[ref-36] Larson P (2016). *Acricoactis brachyacontis* sp. nov. from Adak Island, Alaska, represents a new genus and family of metridioidean sea anemone (Anthozoa: Hexacorallia: Actiniaria). Marine Biodiversity.

[ref-37] Lauretta D, Häussermann V, Brugler MR, Rodríguez E (2014). *Isoparactis fionae* sp. nov. (Cnidaria: Anthozoa: Actiniaria) from Southern Patagonia with a discussion of the family Isanthidae. Organisms Diversity & Evolution.

[ref-39] Martin JP, Garese A, Sar A, Acuña FH (2015). Fouling community dominated by *Metridium senile* (Cnidaria: Anthozoa: Actiniaria) in Bahía San Julián (southern Patagonia, Argentina). Scientia Marina.

[ref-41] Minasian LL (1982). The relationship of size and biomass to fission rate in a clone of the sea anemone, *Haliplanella luciae* (Verrill). Journal of Experimental Marine Biology and Ecology.

[ref-3] Ministry of Environment and Sustainable Development of Argentina (2022). Lista oficial de especies exóticas en Argentina. https://www.argentina.gob.ar/ambiente/biodiversidad/exoticas-invasoras/lista.

[ref-42] Molina LM, Valiñas MS, Pratolongo PD, Elias R, Perillo GME (2009). First record of the sea anemone *Diadumene lineata* (Verrill 1871) associated to *Spartina alterniflora* roots and stems, in marshes at the Bahía Blanca estuary. Argentina Biological Invasions.

[ref-43] Nei M, Kumar S (2000). Molecular evolution and phylogenetics.

[ref-44] Newcomer K, Flenniken MM, Carlton JT (2019). Home and away and home again: discovery of a native reproductive strategy of the globally invading sea anemone *Diadumene lineata* (Verrill, 1869) in a satellite population. Biological Invasions.

[ref-45] Podbielski I, Bock C, Lenz M, Melzner F (2016). Using the critical salinity (S crit) concept to predict invasion potential of the anemone *Diadumene lineata* in the Baltic Sea. Marine Biology.

[ref-46] Riemann-Zürneck K (1975). Actiniaria des Südwestatlantik II. Sagartidae und Metridiidae. Helgoländer Wissenschaftliche Meeresuntersuchungen.

[ref-47] Rodríguez E, Barbeitos M, Daly M, Gusmão LC, Häussermann V (2012). Toward a natural classification: phylogeny of acontiate sea anemones (Cnidaria, Anthozoa, Actiniaria). Cladistics.

[ref-48] Ryan WH, Kubota S (2016). Morphotype distribution of the sea anemone *Diadumene lineata* in Tanabe Bay, Wakayama: a comparison with Uchida, 1936 after 80 years. Publications of the Seto Marine Biological Laboratory.

[ref-49] Ryan WH, Miller TE (2019). Reproductive strategy changes across latitude in a clonal sea anemone. Marine Ecology Progress Series.

[ref-50] Schwindt E, Carlton JT, Orensanz JM, Scarabino F, Bortolus A (2020). Past and future of the marine bioinvasions along the Southwestern Atlantic. Aquatic Invasions.

[ref-51] Shearer TL, van Oppen MJH, Romano SL, Wörheide G (2002). Slow mitochondrial DNA sequence evolution in the Anthozoa (Cnidaria). Molecular Ecology.

[ref-52] Shick JM, Mackie GO (1976). Ecological physiology and genetics of the colonizing actinian *Haliplanella luciae*. Coelenterate Ecology and Behavior.

[ref-53] Shick JM, Lamb SN (1977). Asexual reproduction and genetic population structure in the colonizing sea anemone *Haliplanella luciae*. Biological Bulletin.

[ref-54] Stephenson TA (1935). The british sea anemones.

[ref-55] Tamura K, Stecher G, Kumar S (2021). MEGA11: molecular evolutionary genetics analysis version 11. Molecular Biology and Evolution.

[ref-56] Ting JH, Geller JB (2000). Clonal diversity in introduced populations of an Asian sea anemone in North America. Biological Invasions.

[ref-57] Trifinopoulos J, Nguyen LT, Haeseler A, Minh BQ (2016). W-IQ-TREE: a fast online phylogenetic tool for máximum likelihood analysis. Nucleic Acids Research.

[ref-58] Wood C, Bishop J, Harley J, Mrowicki R (2022). The genome sequence of the orange-striped anemone, *Diadumene lineata* (Verrill, 1869) Welcome. Wellcome Open Research.

[ref-59] Yoshikawa A, Izumi T, Moritaki T, Kimura T, Yanagi K (2022). Carcinoecium-forming sea anemone *Stylobates calcifer* sp. nov. (Cnidaria, Actiniaria, Actiniidae) from the Japanese deep-sea floor: a taxonomical description with its ecological observations. Biological Bulletin.

