# Supplementary material for: Mitochondrial and nuclear gene sequencing confirms the presence of the invasive sea anemone Diadumene lineata (Verrill, 1869) (Cnidaria: Actiniaria) in Argentina
Source: PeerJ. 2023 Nov 27;11:e16479. doi: 10.7717/peerj.16479 (PMC10688303; doi:10.7717/peerj.16479)
Supplement: Supplemental Information 1 [file peerj-11-16479-s001.docx]

**Supplementary Table 1:** Taxa included in this study, with GenBank accession numbers. New data in bold.

| **Name** | **12s** | **16s** | **18s** | **cox3** |
| --- | --- | --- | --- | --- |
| *Acricoactis brachyacontis* | KX451130 | KX451132 | KX451134 | KX451136 |
| *Acricoactis brachyacontis* 1 | KX451131 | KX451133 | KX451135 | KX451137 |
| *Actinauge richardi* | EU190719 | EU190761 | EU190850 | FJ489480 |
| *Actinoscyphia plebeia* | EU190712 | EU190754 | FJ489437 | FJ489476 |
| *Actinothoe sphyrodeta* | FJ489401 | FJ489421 | FJ489440 | FJ489481 |
| *Adamsia palliata* | FJ489398 | FJ489419 | FJ489436 | FJ489474 |
| *Aiptasia couchii* | KP761208 | KP761258 | - | - |
| *Aiptasia mutabilis* | JF832963 | KP761256 | KP761300 | KP761394 |
| *Aiptasia mutabilis* 1 | - | - | KP761304 | KP761404 |
| *Aiptasiogeton hyalinus* | - | KP761257 | KP761294 | KP761409 |
| *Alicia sansibarensis* | KJ482933 | KJ482953 | KJ483016 | KJ483000 |
| *Allantactis parasitica* | FJ489399 | FJ489420 | FJ489439 | FJ489478 |
| *Alvinactis chessi* | GU473278 | GU473296 | GU473312 | GU473352 |
| *Amphianthus sp LG 2009* | FJ489413 | FJ489432 | FJ489450 | FJ489502 |
| *Andvakia boninensis* | EU190717 | EU190759 | EU190848 | FJ489479 |
| *Andvakia discipulorum* | GU473273 | GU473287 | GU473316 | - |
| *Anemonia viridis* | EU190718 | EU190760 | EU190849 | GU473335 |
| *Antholoba achates* | GU473269 | GU473284 | GU473301 | GU473356 |
| *Anthothoe chilensis* | FJ489397 | FJ489416 | FJ489434 | FJ489470 |
| *Antipodactis awii* | GU473271 | GU473286 | GU473303 | GU473337 |
| *Bartholomea annulata* | EU190721 | KP761242 | EU190851 | - |
| *Bathyphellia australis* | FJ489402 | FJ489422 | EF589063 | FJ489482 |
| *Bellactis ilkalyseae* | KR186021 | KR186037 | KR186052 | - |
| *Boloceroides mcmurrichi* | GU473270 | - | EU190852 | KJ483002 |
| *Bunodeopsis globulifera* | KJ482940 | KJ482949 | KJ483025 | KJ482992 |
| *Bunodosoma grande* | EU190722 | EU190765 | EU190853 | GU473336 |
| *Cactosoma sp ER 2010* | GU473279 | GU473297 | GU473313 | GU473346 |
| *Calliactis japonica* | FJ489403 | FJ489423 | FJ489441 | FJ489486 |
| *Calliactis parasitica* | EU190711 | EU190752 | EU190842 | - |
| *Calliactis polypus* | FJ489407 | FJ489427 | FJ489445 | FJ489485 |
| *Calliactis tricolor* | FJ489405 | FJ489425 | FJ489443 | FJ489488 |
| *Cereus herpetodes* | JF832956 | JF832969 | JF832983 | - |
| *Cereus pedunculatus* | EU190724 | EU190767 | EU190855 | FJ489471 |
| *Chondrophellia sp LG 2009* | FJ489406 | FJ489426 | FJ489444 | FJ489489 |
| *Cricophorus nutrix* | - | KT852066 | KT852134 | KT852286 |
| *Cyananthea hourdezi* | GU473275 | GU473293 | GU473309 | GU473364 |
| *Dactylanthus antarcticus* | GU473272 | AY345877 | AF052896 | GU473358 |
| *Diadumene cincta* | EU190725 | EU190769 | EU190856 | FJ489490 |
| *Diadumene leucolena* | JF832957 | JF832977 | JF832986 | JF833006 |
| *Diadumene leucolena* (Brazil) | KY815042 | KY815043 | KY815044 | - |
| *Diadumene lineata* (USA) | EU190730 | EU190774 | EU190860 | FJ489506 |
| *Diadumene lineata* (Japan) | JF832965 | JF832973 | JF832987 | JF833007 |
| *Diadumene paranaensis* | - | KT353112 | - | KT353113 |
| *Diadumene sp* ER 2012 | JF832960 | JF832976 | JF832980 | JF833005 |
| *Diadumene manezinha* (Brazil) | KY815045 | KY815046 | KY815047 | - |
| *Diadumene lineata* 308 (Argentina) | **OP683485** | **OP687085** | **OR395177** | - |
| *Diadumene lineata* 309 (Argentina) | **OP683486** | **OP687086** | **OR395178** | - |
| *Diadumene lineata* 310 (Argentina) | **OP683487** | **OP687087** | **OP688014** | - |
| *Diadumene lineata* MC (Argentina) | **OP683484** | **OP687084** | **OP688013** | - |
| *Diadumene lineata* 2 | MT893227.1 | - | MT895444.1 | - |
| *Exaiptasia diaphana* | KP761188 | KP761239 | KP761284 | KP761385 |
| *Exaiptasia diaphana* 1 | KP761206 | KP761260 | KP761312 | KP761386 |
| *Galatheanthemum profundale* | KJ482919 | KJ482954 | KJ483011 | KJ482978 |
| *Galatheanthemum* sp n MRB 2014 | KJ482918 | KJ482955 | KJ483012 | KJ482977 |
| *Gonactinia prolifera* | KJ482935 | KJ482969 | KJ483008 | KJ482994 |
| *Gonactinia prolifera* 1 | KJ482937 | - | KJ483009 | KJ482995 |
| *Halcampa duodecimcirrata* | JF832966 | EU190776 | AF254375 | - |
| *Halcampoides purpureus* | EU190735 | EU190780 | AF254380 | - |
| *Hormathia armata* | EU190731 | EU190775 | EU190861 | FJ489491 |
| *Hormathia lacunifera* | FJ489409 | FJ489428 | FJ489446 | FJ489492 |
| *Hormathia pectinata* | FJ489415 | FJ489430 | FJ489448 | FJ489497 |
| *Isanthus capensis* | JF832967 | GU473291 | GU473307 | GU473362 |
| *Isoparactis fabiani* | JF832964 | GU473283 | GU473300 | GU473355 |
| *Jasonactis erythraios* | - | GU473289 | GU473305 | GU473339 |
| *Kadosactis antarctica* | FJ489410 | EU190782 | EU190865 | FJ489504 |
| *Laviactis lucida* | KP761192 | KP761243 | - | KP761402 |
| *Metridium senile* | EU190740 | EU190786 | KJ483035 | FJ489494 |
| *Metridium senile* 1 | KJ482916 | KJ482950 | - | KJ482975 |
| *Metridium senile lobatum* | JF832962 | JF832971 | JF832981 | JF833002 |
| *Nemanthus nitidus* | EU190741 | EU190787 | EU190868 | FJ489495 |
| *Ostiactis pearseae* | EU190751 | EU190798 | EU190878 | GU473365 |
| *Paracalliactis* sp LG 2009 | FJ489411 | FJ489429 | FJ489447 | FJ489496 |
| *Paranthus niveus* | GU473277 | GU473295 | GU473311 | GU473344 |
| *Paraphelliactis* sp LG 2009 | FJ489412 | FJ489431 | FJ489449 | FJ489498 |
| *Peronanthus* sp MRB 2014 | KJ482917 | KJ482956 | KJ483014 | KJ482976 |
| *Phellia exlex* | JF832958 | JF832978 | JF832984 | JF833004 |
| *Phellia gausapata* | EU190744 | EU190790 | EU190870 | - |
| *Phymanthus loligo* | EU190745 | EU190791 | EU190871 | GU473345 |
| *Protanthea simplex* | KJ482939 | KJ482970 | KJ483010 | KJ482993 |
| *Sagartia elegans* | - | JF832970 | JF832989 | JF833012 |
| *Sagartia ornata* | JF832959 | JF832975 | JF832985 | JF833011 |
| *Sagartia troglodytes* | EU190746 | EU190792 | EU190872 | FJ489499 |
| *Sagartiogeton laceratus* | EU190748 | EU190794 | EU190874 | FJ489500 |
| *Sagartiogeton undatus* | FJ489400 | FJ489417 | FJ489435 | FJ489472 |
| *Telmatactis* sp ER 2012 | JF832968 | JF832979 | KJ483013 | - |
| *Verrillactis paguri* | FJ489414 | FJ489433 | - | FJ489503 |
