# Supplementary material for: Mitochondrial and nuclear gene sequencing confirms the presence of the invasive sea anemone Diadumene lineata (Verrill, 1869) (Cnidaria: Actiniaria) in Argentina
Source: PeerJ. 2023 Nov 27;11:e16479. doi: 10.7717/peerj.16479 (PMC10688303; doi:10.7717/peerj.16479)
Supplement: Supplemental Information 2 [file peerj-11-16479-s002.docx]

**Supplemental Information 2:** K2P model for 12S, 16S, 18S and 28S.

Comparison of the K2P distances of the 12S mitochondrial marker between the sequences of *Diadumene lineata* with other congeneric species.

| **12S** | **1** | **2** | **3** | **4** | **5** | **6** | **7** | **8** | **9** | **10** | **11** | **12** |
| --- | --- | --- | --- | --- | --- | --- | --- | --- | --- | --- | --- | --- |
| *D. lineata*_Mar Chiquita |  |  |  |  |  |  |  |  |  |  |  |  |
| *D. lineata* 308 Garipe Beach | 0.0000 |  |  |  |  |  |  |  |  |  |  |  |
| *D. lineata* 309 Garipe Beach | 0.0000 | 0.0000 |  |  |  |  |  |  |  |  |  |  |
| *D. lineata* 310 Garipe Beach | 0.0000 | 0.0000 | 0.0000 |  |  |  |  |  |  |  |  |  |
| *D. lineata* (USA) EU190730.1 | 0.0000 | 0.0000 | 0.0000 | 0.0000 |  |  |  |  |  |  |  |  |
| *D. lineata* (Japan) JF832965.1 | 0.0000 | 0.0000 | 0.0000 | 0.0000 | 0.0000 |  |  |  |  |  |  |  |
| *D. lineata* MT893227.1 | 0.0000 | 0.0000 | 0.0000 | 0.0000 | 0.0000 | 0.0000 |  |  |  |  |  |  |
| *Diadumene* sp. JF832960.1 | 0.0096 | 0.0096 | 0.0096 | 0.0096 | 0.0096 | 0.0096 | 0.0096 |  |  |  |  |  |
| *D. cincta* EU190725.1 | 0.0096 | 0.0096 | 0.0096 | 0.0096 | 0.0096 | 0.0096 | 0.0096 | 0.0000 |  |  |  |  |
| *D. manezinha* KY815045.1 | 0.0057 | 0.0057 | 0.0057 | 0.0057 | 0.0057 | 0.0057 | 0.0057 | 0.0154 | 0.0154 |  |  |  |
| *D. leucolena* JF832957.1 | 0.0057 | 0.0057 | 0.0057 | 0.0057 | 0.0057 | 0.0057 | 0.0057 | 0.0154 | 0.0154 | 0.0000 |  |  |
| *D. leucolena* KY815042.1 | 0.0057 | 0.0057 | 0.0057 | 0.0057 | 0.0057 | 0.0057 | 0.0057 | 0.0154 | 0.0154 | 0.0000 | 0.0000 |  |

Comparison of the K2P distances of the 16S mitochondrial marker between the sequences of *Diadumene lineata* with other congeneric species.

| **16S** | **1** | **2** | **3** | **4** | **5** | **6** | **7** | **8** | **9** | **10** | **11** | **12** |
| --- | --- | --- | --- | --- | --- | --- | --- | --- | --- | --- | --- | --- |
| *D. lineata*_Mar Chiquita |  |  |  |  |  |  |  |  |  |  |  |  |
| *D. lineata* 308 Garipe Beach | 0.0000 |  |  |  |  |  |  |  |  |  |  |  |
| *D. lineata* 309 Garipe Beach | 0.0000 | 0.0000 |  |  |  |  |  |  |  |  |  |  |
| *D. lineata* 310 Garipe Beach | 0.0000 | 0.0000 | 0.0000 |  |  |  |  |  |  |  |  |  |
| *D. lineata* (USA) EU190774.1 | 0.0000 | 0.0000 | 0.0000 | 0.0000 |  |  |  |  |  |  |  |  |
| *D. lineata* (Japan) JF832973.1 | 0.0000 | 0.0000 | 0.0000 | 0.0000 | 0.0000 |  |  |  |  |  |  |  |
| *Diadumene sp.* JF832976.1 | 0.0179 | 0.0179 | 0.0179 | 0.0179 | 0.0179 | 0.0179 |  |  |  |  |  |  |
| *D. cincta* EU190769.1 | 0.0179 | 0.0179 | 0.0179 | 0.0179 | 0.0179 | 0.0179 | 0.0000 |  |  |  |  |  |
| *D. paranaensis* KT353112.1 | 0.0210 | 0.0210 | 0.0210 | 0.0210 | 0.0210 | 0.0210 | 0.0029 | 0.0029 |  |  |  |  |
| *D. manezinha* KY815046.1 | 0.0179 | 0.0179 | 0.0179 | 0.0179 | 0.0179 | 0.0179 | 0.0334 | 0.0334 | 0.0366 |  |  |  |
| *D. leucolena* JF832977.1 | 0.0149 | 0.0149 | 0.0149 | 0.0149 | 0.0149 | 0.0149 | 0.0303 | 0.0303 | 0.0334 | 0.0029 |  |  |
| *D. leucolena* KY815043.1 | 0.0149 | 0.0149 | 0.0149 | 0.0149 | 0.0149 | 0.0149 | 0.0303 | 0.0303 | 0.0334 | 0.0029 | 0.0000 |  |

Comparison of the K2P distances of the 18S nuclear marker between the sequences of *Diadumene lineata* with other congeneric species.

| **18S** | **1** | **2** | **3** | **4** | **5** | **6** | **7** | **8** | **9** | **10** |
| --- | --- | --- | --- | --- | --- | --- | --- | --- | --- | --- |
| *D. lineata*_Mar Chiquita |  |  |  |  |  |  |  |  |  |  |
| *D. lineata* (USA) EU190860.1 | 0.0000 |  |  |  |  |  |  |  |  |  |
| *D. lineata* (Japan) JF832987.1 | 0.0000 | 0.0000 |  |  |  |  |  |  |  |  |
| *D. lineata* MT895444.1 | 0.0000 | 0.0000 | 0.0000 |  |  |  |  |  |  |  |
| *D. lineata* 310 Garipe Beach | 0.0074 | 0.0074 | 0.0074 | 0.0074 |  |  |  |  |  |  |
| *D. cincta* EU190856.1 | 0.0425 | 0.0425 | 0.0425 | 0.0425 | 0.0503 |  |  |  |  |  |
| *Diadumene* sp. JF832980.1 | 0.0425 | 0.0425 | 0.0425 | 0.0425 | 0.0503 | 0.0000 |  |  |  |  |
| *D. manezinha* KY815047.1 | 0.0432 | 0.0432 | 0.0432 | 0.0432 | 0.0511 | 0.0345 | 0.0345 |  |  |  |
| *D. leucolena* JF832986.1 | 0.0425 | 0.0425 | 0.0425 | 0.0425 | 0.0504 | 0.0331 | 0.0331 | 0.0027 |  |  |
| *D. leucolena* KY815044.1 | 0.0432 | 0.0432 | 0.0432 | 0.0432 | 0.0511 | 0.0338 | 0.0338 | 0.0020 | 0.0006 |  |

Comparison of the K2P distances of the 28S nuclear marker between the sequences of *Diadumene lineata* with other congeneric species.

| **28S** | **1** | **2** | **3** | **4** | **5** | **6** | **7** | **8** | **9** | **10** | **11** | **12** |
| --- | --- | --- | --- | --- | --- | --- | --- | --- | --- | --- | --- | --- |
| *D. lineata*_Mar Chiquita |  |  |  |  |  |  |  |  |  |  |  |  |
| *D. lineata* (USA) EU190819.1 | 0.0000 |  |  |  |  |  |  |  |  |  |  |  |
| *D. lineata* KJ483108.1 | 0.0000 | 0.0000 |  |  |  |  |  |  |  |  |  |  |
| *D. lineata* KJ483107.1 | 0.0000 | 0.0000 | 0.0000 |  |  |  |  |  |  |  |  |  |
| *D. lineata* (Japan) JF832998.1 | 0.0000 | 0.0000 | 0.0000 | 0.0000 |  |  |  |  |  |  |  |  |
| *D. lineata* MT893020.1 | 0.0045 | 0.0045 | 0.0045 | 0.0045 | 0.0045 |  |  |  |  |  |  |  |
| *D. cincta* KJ483106.1 | 0.1374 | 0.1374 | 0.1374 | 0.1374 | 0.1374 | 0.1373 |  |  |  |  |  |  |
| *D. cincta* EU190814.1 | 0.1294 | 0.1294 | 0.1294 | 0.1294 | 0.1294 | 0.1293 | 0.0254 |  |  |  |  |  |
| *Diadumene* sp. JF832990.1 | 0.1137 | 0.1137 | 0.1137 | 0.1137 | 0.1137 | 0.1136 | 0.0277 | 0.0184 |  |  |  |  |
| *Diadumene* sp. KJ483130.1 | 0.1137 | 0.1137 | 0.1137 | 0.1137 | 0.1137 | 0.1136 | 0.0277 | 0.0184 | 0.0000 |  |  |  |
| *D. leucolena* KJ483123.1 | 0.0928 | 0.0928 | 0.0928 | 0.0928 | 0.0928 | 0.0980 | 0.0922 | 0.0820 | 0.0672 | 0.0672 |  |  |
| *D. leucolena* JF832995.1 | 0.0928 | 0.0928 | 0.0928 | 0.0928 | 0.0928 | 0.0980 | 0.0922 | 0.0820 | 0.0672 | 0.0672 | 0.0000 |  |
